# Joint EANM/EANO/RANO practice guidelines/SNMMI procedure standards for imaging of gliomas using PET with radiolabelled amino acids and [^18^F]FDG: version 1.0

**DOI:** 10.1007/s00259-018-4207-9

**Published:** 2018-12-05

**Authors:** Ian Law, Nathalie L. Albert, Javier Arbizu, Ronald Boellaard, Alexander Drzezga, Norbert Galldiks, Christian la Fougère, Karl-Josef Langen, Egesta Lopci, Val Lowe, Jonathan McConathy, Harald H. Quick, Bernhard Sattler, David M. Schuster, Jörg-Christian Tonn, Michael Weller

**Affiliations:** 1 Department of Clinical Physiology, Nuclear Medicine and PET, Rigshospitalet, University of Copenhagen, 9, Blegdamsvej, 2100-DK Copenhagen Ø, Denmark; 2 Department of Nuclear Medicine, Ludwig-Maximilians-University, Munich, Germany; 3 Department of Nuclear Medicine, Clínica Universidad de Navarra, University of Navarre, Pamplona, Spain; 4 Department of Nuclear Medicine and Molecular Imaging, University Medical Center Groningen, Groningen, The Netherlands; 5 Department of Radiology and Nuclear Medicine, VU University Medical Center, Amsterdam, The Netherlands; 6 Department of Nuclear Medicine, University Hospital Cologne, Cologne, Germany; 7 Department of Neurology, University Hospital Cologne, Cologne, Germany; 8 Institute of Neuroscience and Medicine (INM-3, -4), Forschungszentrum Julich, Julich, Germany; 9 Division of Nuclear Medicine and Clinical Molecular Imaging, Department of Radiology, University of Tübingen, Tübingen, Germany; 10 Department of Nuclear Medicine, RWTH University Aachen, Aachen, Germany; 11 Department of Nuclear Medicine, Humanitas Clinical and Research Hospital, Rozzano, Italy; 12 Department of Radiology, Nuclear Medicine, Mayo Clinic, Rochester, MN USA; 13 Division of Molecular Imaging and Therapeutics, University of Alabama at Birmingham, Birmingham, AL USA; 14 High-Field and Hybrid MR Imaging, University Hospital Essen, Essen, Germany; 15 Department for Nuclear Medicine, University Hospital Leipzig, Leipzig, Germany; 16 Division of Nuclear Medicine and Molecular Imaging, Department of Radiology and Imaging Sciences, Emory University, Atlanta, GA USA; 17 Department of Neurosurgery, Ludwig-Maximilians-University Munich, Munich, Germany; 18 Department of Neurology, University Hospital Zurich, Zurich, Switzerland

**Keywords:** FDG, FET, MET, FDOPA, PET, Glioma

## Abstract

These joint practice guidelines, or procedure standards, were developed collaboratively by the European Association of Nuclear Medicine (EANM), the Society of Nuclear Medicine and Molecular Imaging (SNMMI), the European Association of Neurooncology (EANO), and the working group for Response Assessment in Neurooncology with PET (PET-RANO). Brain PET imaging is being increasingly used to supplement MRI in the clinical management of glioma. The aim of these standards/guidelines is to assist nuclear medicine practitioners in recommending, performing, interpreting and reporting the results of brain PET imaging in patients with glioma to achieve a high-quality imaging standard for PET using FDG and the radiolabelled amino acids MET, FET and FDOPA. This will help promote the appropriate use of PET imaging and contribute to evidence-based medicine that may improve the diagnostic impact of this technique in neurooncological practice. The present document replaces a former version of the guidelines published in 2006 (Vander Borght et al. Eur J Nucl Med Mol Imaging. 33:1374–80, 2006), and supplements a recent evidence-based recommendation by the PET-RANO working group and EANO on the clinical use of PET imaging in patients with glioma (Albert et al. Neuro Oncol. 18:1199–208, 2016). The information provided should be taken in the context of local conditions and regulations.

## Preamble

The Society of Nuclear Medicine and Molecular Imaging (SNMMI) is an international scientific and professional organization founded in 1954 to promote the science, technology, and practical application of nuclear medicine. Its 18,000 members are physicians, technologists and scientists specializing in the research and practice of nuclear medicine. In addition to publishing journals, newsletters and books, the SNMMI also sponsors international meetings and workshops designed to increase the competencies of nuclear medicine practitioners and to promote new advances in the science of nuclear medicine. The European Association of Nuclear Medicine (EANM) is a professional nonprofit medical association that facilitates communication worldwide among individuals pursuing clinical and research excellence in nuclear medicine, and had 2,800 members in 2017. The EANM was founded in 1985.

The SNMMI/EANM will periodically define new standards/guidelines for nuclear medicine practice to help advance the science of nuclear medicine and to improve the quality of service to patients. Existing standards/guidelines will be reviewed for revision or renewal, as appropriate, on their fifth anniversary or sooner, if indicated. As of February 2014, the SNMMI guidelines will now be referred to as procedure standards. Any previous practice guidelines or procedure guidelines that describe how to perform a procedure are now considered SNMMI procedure standards. Each of the standards/guidelines, that represents a policy statement by the SNMMI/EANM, has undergone a thorough consensus process in which it has been subjected to extensive review. The SNMMI/EANM recognizes that the safe and effective use of diagnostic nuclear medicine imaging requires specific training, skills and techniques, as described in each document.

The EANM and SNMMI have written and approved these standards/guidelines to promote the use of nuclear medicine procedures of high quality. These standards/guidelines are intended to assist practitioners in providing appropriate nuclear medicine care for patients. They are not inflexible rules or requirements of practice and are not intended, nor should they be used, to establish a legal standard of care. For these reasons and those set forth below, the SNMMI/EANM cautions against the use of these standards/guidelines in litigation in which the clinical decisions of a practitioner are called into question.

The ultimate judgment regarding the propriety of any specific procedure or course of action must be made by medical professionals taking into account the unique circumstances of each case. Thus, there is no implication that an approach differing from the standards/guidelines, standing alone, is below the standard of care. To the contrary, a conscientious practitioner may responsibly adopt a course of action different from that set forth in the standards/guidelines when, in the reasonable judgment of the practitioner, such course of action is indicated by the condition of the patient, limitations of available resources, or advances in knowledge or technology subsequent to publication of the standards/guidelines.

The practice of medicine involves not only the science but also the art of dealing with the prevention, diagnosis, alleviation and treatment of disease. The variety and complexity of human conditions make it impossible to always reach the most appropriate diagnosis or to predict with certainty a particular response to treatment. Therefore, it should be recognized that adherence to these standards/guidelines will not ensure an accurate diagnosis or a successful outcome. All that should be expected is that the practitioner will follow a reasonable course of action based on current knowledge, available resources and the needs of the patient to deliver effective and safe medical care. The sole purpose of these standards/guidelines is to assist practitioners in achieving this objective.

The present guidelines/standards were developed collaboratively by the EANM and SNMMI with the European Association of Neurooncology (EANO) and the working group for Response Assessment in Neurooncology with PET (PET-RANO). They summarize the views of the Neuroimaging, Oncology and Physics Committees of the EANM, the Brain Imaging Council of the SNMMI, the EANO, and PET-RANO, and reflect recommendations for which the EANM cannot be held responsible. The recommendations should be taken into the context of good practice of nuclear medicine and do not substitute for national and international legal or regulatory provisions.

## Introduction

Gliomas are the second most common primary brain tumour with an annual incidence rate of around six cases per 100,000 individuals worldwide [1]. Gliomas represent approximately 27% of all central nervous system (CNS) tumours and 80% of malignant CNS tumours, and are a leading cause of cancer mortality in adults. The most common of all malignant brain and CNS tumours is glioblastoma (46%) which is associated with a median overall survival of 15 months in patients treated with maximal safe tumour resection, concomitant radiotherapy/chemotherapy and adjuvant chemotherapy [2].

Magnetic resonance imaging (MRI) is the primary clinical imaging modality in patients with glioma at all disease stages including the primary evaluation, presurgical planning, early postsurgical evaluation of residual tumour, radiotherapy planning, surveillance during chemotherapy, and definition of recurrence.

There are defined objective and standardized MRI-based criteria for response assessment in neurooncology (RANO) applied to clinical trials in patients with brain tumours. However, MRI contrast enhancement can be unreliable as a surrogate for tumour size or growth. It nonspecifically reflects vascular surface area and the permeability of the contrast agent across a disrupted blood–tumour barrier and may represent tumour biology or a number of other factors including therapy-induced inflammation. Contrast enhancement can be influenced by therapeutics that affect tumour vascular permeability, such as corticosteroid, antiangiogenic [3] or immunotherapy agents [4]. Because of the growing awareness of significant limitations of MRI in the management of glioma, the RANO criteria were recently updated [5]. Hence, to identify infiltrative glioma tissue, the RANO definition of tumour progression was supplemented by inclusion of “significant” enlarging areas of nonenhancing tumour on MRI T2-weighted and fluid-attenuated inversion recovery (FLAIR) image sequences. However, precise quantification of increases in the T2/FLAIR signal could not be defined and other causes of increased T2 or FLAIR signal, such as radiation effects, demyelination, ischaemic injury and oedema have to be considered in the evaluation of progression.

Molecular imaging using positron emission tomography (PET) is a well-established method in systemic oncology [6], and is being increasingly used to supplement MRI in the clinical management of glioma [7–9]. PET imaging could have an important role in clinical trials of new strategies for the treatment of glioma, e.g. immunotherapy, where pseudoprogression is particularly challenging for MRI [4]. Recently an evidence-based recommendation by the PET-RANO working group and EANO on the clinical use of PET imaging in gliomas has been published focusing on radiotracers used in clinical practice imaging, i.e. glucose metabolism, 2-deoxy-2-[^18^F]fluoro-D-glucose (FDG), and system L amino acid transport comprising [^11^C-methyl]-methionine (MET), *O*-(2-[^18^F]fluoroethyl)-L-tyrosine (FET) and 3,4-dihydroxy-6-[^18^F]fluoro-L-phenylalanine (FDOPA) [7].

The present guidelines/standards focus on the technical aspects of PET image acquisition with the above-mentioned radiotracers, and thus replace all previously published guidelines on glioma imaging [10].

## Aim

The aim of these standards/guidelines is to assist nuclear medicine practitioners in recommending, performing, interpreting, and reporting the results of brain PET imaging in patients with glioma.

## Definitions

1. PET systems provide static, dynamic or gated images of the distribution of positron-emitting radionuclides within the body by detecting pairs of photons produced in coincidence by the annihilation of a positron and an electron. PET images are produced by a reconstruction process using the coincidence pair data.
2. PET is generally combined with computed tomography (CT) in a single system (PET/CT). Combined PET/MRI systems are also available for clinical use but are currently less widely available.
3. Nuclear medicine computer systems and software applications collect, quantitate, analyse and display the imaging information.

## Common clinical indications

Common indications for PET imaging in glioma include, but are not limited to, the following [7]:

1. At primary diagnosis
  - Differentiation of grade III and IV tumours from nonneoplastic lesions or grade I and II gliomas
  - Prognostication of gliomas
  - Definition of the optimal biopsy site (e.g. site of maximum tracer uptake)
  - Delineation of tumour extent for surgery and radiotherapy planning
2. Diagnosis of tumour recurrence
  - Differentiation of glioma recurrence from treatment-induced changes, e.g. pseudoprogression, radionecrosis
3. Disease and therapy monitoring
  - Detection of malignant transformation in grade I and II gliomas
  - Response assessment during and after radiotherapy and/or chemotherapy
  - Differentiation of tumour response from pseudoresponse during antiangiogenic therapy

The performances of the PET tracers presented in this guideline are different as discussed in a recent evidence-based recommendation [7].

FDG PET plays a more limited role than amino acid PET in the imaging of gliomas due to the high physiological uptake of FDG in normal brain grey matter and variable uptake by inflammatory lesions. FDG PET is most often used to distinguish tumour recurrence from radiation necrosis in enhancing brain lesions or to distinguish glioma from CNS lymphoma or opportunistic infection.

## Qualifications and responsibilities of personnel

### Physician

PET examinations should be performed by, or under the supervision of, a physician specialized in nuclear medicine and certified by accreditation boards. In Europe, the certified nuclear medicine physician who performed the study and signed the report is responsible for the procedure, according to national laws and rules. In the United States, the SNMMI Procedure Guideline for General Imaging (Society of Nuclear Medicine, 2010. http://snmmi.files.cms-plus.com/docs/General_Imaging_Version_6.0.pdf) should be consulted.

### Technologist

PET examinations should be performed by a qualified registered/certified nuclear medicine technologist. The following documents should be consulted: Performance Responsibility and Guidelines for Nuclear Medicine Technologists 3.1 and the EANM Benchmark Document Nuclear Medicine Technologists’ Competencies (http://www.eanm.org/content-eanm/uploads/2016/11/EANM_2017_TC_Benchmark.pdf [11]). In some jurisdictions, additional qualifications may be necessary for technologists to operate the CT or MR components.

### Physicist

PET examinations should be performed using PET systems that comply with national or international quality standards (*see* section Quality control and improvement). A certified clinical physicist is responsible for ensuring that PET systems meet these quality standards. Moreover, in some countries it is required that a board-certified medical physicist be available to advise the above-mentioned personnel in running the imaging systems and/or in managing dysfunction or failure of the systems, and to perform the examinations. In addition, examinations should be performed following national or international dosimetry and radiation safety standards in relation to both patients and personnel.

## Examination procedures/specifications

Recommendations for FDG-specific procedures are defined in previous guidelines, and only recommendations that are new or particular to glioma are discussed [12, 13].

### Request

A nuclear medicine imaging facility staff member should check with the nuclear pharmacy provider as to the availability of the radiotracer before scheduling the examination. Advanced notice may be required for tracer delivery.

The study requisition should include:

1. Appropriate clinical information about the patient and a clearly specified clinical question to justify the study and to allow appropriate examination/study coding (*see* section Common clinical indications).
2. Information about the ability of the patient to cooperate with the examination and the participation of a carer may be helpful.
3. Information about current medications, including glucocorticoids, for correct study interpretation and to avoid unwanted pharmacological interaction effects if mild sedation is necessary.
4. History of prior therapy, including prior chemotherapy, surgery and radiotherapy, which might affect radiopharmaceutical distribution.
5. Results of pertinent imaging studies, resections and biopsies performed, and laboratory results.
6. For PET/MRI, all patients should be screened at request for relevant contraindications to MRI using a standardized checklist (e.g. pregnancy, contrast agent reactions, implants, ports, catheters, metallic implants, vascular stents, coils, active implants, cardiac pacemakers, claustrophobia, etc.) [14].

### Patient preparation and precautions

1. The height and body weight of the patient must be documented for measurement of standardized uptake values (SUV; *see* section Static FET, MET, FDOPA PET, item 1).
2. Recent morphological imaging with MRI (T1, T1 + contrast medium, T2/FLAIR) should be available for image fusion.
3. The patient should be informed about the procedure to guarantee optimal compliance.
4. The patient should be able to lie down quietly for at least 30 to 40 min.
5. If sedation is required for MET, FET or FDOPA imaging, it should start about 20–60 min before the examination. If sedation is required for FDG imaging, sedation should start as late as possible after FDG administration, ideally at least 30 min after FDG injection but prior to imaging.
6. The patient should be required to fast before the examination to ensure stable metabolic conditions. A minimum 4-h fast is recommended for MET, FET, FDOPA, and FDG imaging.
7. Serum glucose should/may be measured before FDG administration so that the interpreting physician is aware of the potential for altered biodistribution.
8. Before scanning, patients should empty their bladder for maximum comfort during the study and in order to reduce the absorbed dose to the bladder (*see* section Radiation safety).
9. In pregnant patients, it is necessary to make a clinical decision that weighs the benefits to the patient against possible harm [6].
10. For FDOPA PET, premedication with carbidopa is not necessary. Most published studies to date with FDOPA PET in patients with brain tumour have not used carbidopa or other inhibitors of peripheral FDOPA metabolism.
11. If the PET study is to be acquired as part of a PET/MRI study:
  - Check MRI contraindications from checklist (*see* section Request, item 6).
  - Remove all metal from the patient (e.g. dental prostheses, clothing with zippers and buttons), and provide cotton clothing for the patient.
  - In patients with an implant, the specific type of implant, its location and its component materials need to be known before an MRI examination. The patient should be asked for an implant pass. The safety level of an implant/device should be checked with the manufacturer (e.g. online): “MRI unsafe” is an absolute contraindication; “MRI conditional” is a relative contraindication, conditions apply; “MRI safe” is no contraindication.
  - If the patient has a metal implant or active device labelled “MRI conditional”, information should be obtained about all the conditions that may apply for safe MRI examination (e.g. from the implant pass or online).
  - Beyond safety concerns, implants may cause artefacts, large-volume signal voids and geometric distortions on MRI images. These may hamper image reading.
12. It is recommended that the patient stay well hydrated and empty the bladder often.

### Radiopharmaceuticals

- 2-Deoxy-2-[^18^F]fluoro-D-glucose; fludeoxyglucose F18 (FDG)
- *O*-(2-[^18^F]Fluoroethyl)-L-tyrosine (FET)
- L-[methyl-^11^C]Methionine; methionine C11 (MET)
- 3,4-Dihydroxy-6-[^18^F]fluoro-L-phenylalanine; fluorodopa F18 (FDOPA)

#### Preparation of radiopharmaceuticals

All radiopharmaceuticals must be prepared by qualified personnel according to cGMP-compliant methods that conform to regulatory requirements. The radiopharmaceutical is delivered ready to use. Quality control (QC) is carried out by the manufacturer prior to delivery of the final product.

#### Administered activity in adults

The recommended injected activities for brain imaging in adults are as follows:

- ^18^F-FET: 185–200 MBq
- ^11^C-MET: 370–555 MBq
- ^18^F-FDOPA: 185–200 MBq
- ^18^F-FDG: 185–200 MBq

#### Administered activity in children

In children, the radioactivity dose should be calculated as a fraction of the dose for adults according to the child’s body weight using the factors provided by the EANM Paediatric Task Group [15]. The administered dose may be reduced in systems with higher sensitivity (*see* section Radiation safety).

The above radiopharmaceuticals should be injected as a bolus.

### PET acquisition protocols

#### Positioning

The scan should be performed with the patient positioned with the head in a dedicated holder and the arms along the body. The entire brain should be in the field of view, including the entire cerebellum, and extreme neck extension or flexion should be avoided.

#### Head stability

1. The patient should be instructed immediately before the PET acquisition to avoid head movements during all parts of the investigation.
2. Head stability can be achieved by positioning the patient comfortably with the head secured as completely as possible in the dedicated holder. Tape, padding or other flexible head restraints including a thermoplastic mould and vacuum mattress for children may be employed and are often helpful particularly for radiotherapy planning.
3. During the entire investigation, the patient should be continuously visually monitored. Monitoring is particularly important in patients with tumour-associated seizures. Seizure activity during the uptake phase of FDG and FET can lead to increased uptake in the brain affected by seizure activity and can be mistaken for tumour.

#### PET imaging sequence

The preferred PET imaging sequence is:

1. CT scout topogram for PET/CT to set up the field of view.
2. For attenuation correction (*see* section Attenuation correction below), a low-dose CT scan, MRI attenuation correction scan or transmission scan should be performed. Mathematical attenuation correction (i.e. based on the patient’s external contour (derived from a PET image without attenuation correction) should not be applied.
3. Static or dynamic single field of view PET acquisition.

#### Attenuation correction

Images should be acquired in 3D data acquisition mode and attenuation correction should be based on a low-dose CT scan, MRI attenuation correction scan, or 511-keV transmission scan. If a 511-keV transmission scan is used, the transmission images should be acquired before tracer injection. CT parameters should always be chosen to ensure the lowest doses that are compatible with this purpose are administered to the patient.

The following points relevant to attenuation correction in PET/MRI are to be considered:

1. In PET/MRI, attenuation correction is based on MRI imaging. Thus, MRI-based attenuation correction needs to be accurate and free from artefacts to provide accurate PET quantification.
2. The latest version of MRI attenuation-correction software should be used, including ultrashort echo time (UTE), zero TE (ZTE) sequences, or bone-models for bone detection in brain PET/MRI, where available [16, 17].
3. Various attenuation correction strategies for PET/MRI have been implemented. Some may lead to systematic differences in the activity distribution and calculated semiquantitative metrics (*see* section Static FET, MET, FDOPA PET) [18] that should carefully be considered during interpretation of PET images [19, 20].
4. MRI attenuation-corrected images must routinely be checked for artefacts, consistency and plausibility during PET/MRI reading. Artefacts on MRI attenuation correction have a direct effect on PET quantification in brain PET/MRI. Typically artefacts may arise from mis-segmentation of brain/fat/bone tissue, or from metallic dental prostheses and metallic implants such as coils, stents, surgical clips, etc. [18, 21, 22]. Artefacts may show as signal voids that exceed the true dimensions of the metal inclusions. Thus, most artefacts are readily detectable on MRI attenuation-corrected images and indicate regions of potentially inaccurate PET quantification [23, 24].
5. Where applicable, time-of-flight PET detection should be used to reduce the impact of metal artefacts in brain PET/MRI examinations [25].
6. Only radiofrequency (RF) head coils labelled for combined PET/MRI use only should be used. Using standard RF head coils labelled for MRI-only use will not be considered in PET/MRI attenuation correction and their use may thus lead to inaccurate PET quantification and artefacts on PET.
7. In longitudinal studies, the patient should always be scanned on the same system using the same procedures to avoid changes related to differences in imaging technology or methodology. In cross-sectional studies, possible differences among scans related to the technologies being used should be considered. Particular care is needed in paediatric patients.

#### PET comparability

To ensure PET comparability, a standardized protocol for clinical reading should be used with a fixed time for the start of image acquisition.

1. FET: 20 min static image acquisition obtained 20 min after injection. This may be part of a 40–50 min dynamic image acquisition initiated at tracer injection. Dynamic image acquisitions should be started using short frames that progressively increase in duration. From 10 to 50 min after injection 5-min acquisition frames should be used to allow the tracer uptake slope to be assessed during this interval. To allow precise information on the tracer uptake phase to be obtained, the following image acquisition frame sequences could be used during the first 10 min after injection: 12 frames of 5 s, 6 frames of 10 s, 6 frames of 30 s and 5 frames of 60 s.
2. MET: 20 min static image acquisition obtained 10 min after injection.
3. FDOPA: 10–20 min static image acquisition obtained 10–30 min after injection.
4. FDG: 10–20 min static image acquisition obtained at least 45 min after injection.

#### Movement artefacts

If movement artefacts are expected, it may be helpful to acquire the static time window dynamically, e.g. in 5-min frames, or in list mode. Check the sinograms, and use only those of the properly acquired motion-free time period for reconstruction.

### PET image reconstruction

1. During image reconstruction, all corrections for quantitative interpretation are required including attenuation, scatter, random, dead time and decay corrections, as well as detector sensitivity normalization.
2. Time of flight acquisitions and reconstructions are allowed, although the benefit for brain imaging has not yet been fully investigated.
3. Iterative reconstruction is the field standard and should be applied. However, if iterative reconstruction would result in upward bias due to non-negativity constraints applied during reconstruction, filtered back-projection reconstruction may be used as an alternative method.
4. The use of resolution modelling during reconstruction, so-called point-spread-function (PSF) reconstructions, may give rise to Gibbs artefacts and quantitative errors [26], and this method is not recommended.
5. To harmonize PET image quality, the following reconstruction settings/protocols are recommended:
  - One of the reconstructions should be performed using settings such that the reconstructed images meet EARL requirements for image quality recovery [6], thereby allowing harmonization of PET data for multicentre settings or for use with reference datasets.
  - As the above harmonizing reconstruction settings will ensure comparable image quality among different generations of PET/CT systems, a higher resolution reconstruction may be desired or required for visual interpretation or tumour delineation. When a specific PET system allows the use of multiple reconstructions, a high-resolution dedicated brain reconstruction protocol may be applied. Such a protocol should preferably meet the following requirements:
    Voxels size 1–2 mm, but <3 mm in any direction
    Reconstructed spatial resolution <6 mm full-width at half-maximum

### Interpretation/quantification

#### Standardized uptake value calculations and image analysis

##### General image display

1. PET images should have pixels of at least 16 bits to provide an adequate range of values, and appropriate image scaling should be employed for image display. A colour scale may be used. PET images should be displayed in the transaxial orientation and additionally correlated with morphological images in the coronal and sagittal planes.
2. Internal landmarks can be used for reorientation to achieve a standardized image display. Reorientation procedures based on the intercommissural line are commonly used [27].
3. FET, MET, FDOPA: If the display scale is in colour, it should be adjusted so that the background radioactivity of healthy brain is in the lower third of the range (blue hue in the widely used Sokoloff scale), in order to create standardized conditions for the visual detection of increased tracer accumulation above background.
4. FDG: The display scale should be initially adjusted so that the radioactivity in normal cerebral cortex is near the maximum of the scale. If lesions have higher uptake than the cerebral cortex, the scale should be adjusted such that the lesion with the highest uptake is near to the maximum of the scale. Colour scales with 10 or 20 increments are useful to estimate the relative concentrations of FDG across brain regions and in lesions.

##### Static FET, MET, FDOPA PET

1. Calculation of the SUV is optional and may be performed by dividing the radioactivity concentration (in kilobecquerels per millilitre) in the tissue by the radioactivity (in megabecquerels) injected per body weight (in kilograms), body surface area (in metres squared) or lean body mass (in kilograms), depending on the most appropriate distribution volume for each tracer.
2. Standard summation images in the ranges defined in section PET comparability (items 1, 2 and 3) are used for clinical reading and should be coregistered and fused with recent high-resolution contrast-enhanced T1-weighted, T2-weighted/FLAIR MRI sequences. Fusion with other MRI sequences is optional. Usually vendor-provided coregistration software solutions are sufficiently robust for clinical use, but must be routinely checked for misalignment during reading. This can be done by adjusting the PET colour scale to clearly visualize the scalp and nose of the patient so that these structures can be compared between the PET and MRI images.
3. In a first visual analysis, qualitative evaluation can be performed and the lesion of interest can be classified as either *positive*, when tracer uptake visually exceeds the background activity in the contralateral cortex, or *negative*, when no increased uptake can be seen.
4. MET, FET: In order to ensure intraindividual as well as interindividual comparability, semiquantitative measures of mean and maximal tumour activity uptake values can be calculated as ratios in relation to healthy appearing reference brain tissue (tumour to background ratios, TBRmean and TBRmax, respectively).
  - The mean physiological brain activity uptake in healthy appearing cortex of the hemisphere contralateral to the tumour including grey and white matter is measured from a large “banana”/crescent-shaped background volume of interest (VOI) [28].
  - The measurement of TBRmean depends on the delineation of the tumour VOI.
5. FDOPA: Semiquantitative measures of mean and maximal tumour activity uptake values can be calculated as ratios in relation to healthy appearing striatum [29] contralateral to the tumour (tumour to striatum ratios, TSRmean and TSRmax, respectively). The striatum is the most commonly used reference region. Other reference regions have not been investigated systematically.

##### Static FDG PET

1. A static image acquired for 10–20 min at least 45 min after injection is used for clinical FDG PET reading, and should be coregistered and fused with recent high-resolution contrast-enhanced T1 and T2/FLAIR MRI sequences. Fusion with other MRI sequences is optional.
2. SUVs used directly are generally of limited value in the clinical interpretation of FDG PET neurooncology studies.
3. Qualitative visual analysis can be performed and the lesion of interest can be classified as either *positive*, when FDG uptake visually exceeds the activity in a reference region (e.g. normal appearing white matter or cerebral cortex), or *negative*, when FDG uptake in the lesion is less than that in the reference region. Using white matter as the reference region provides better sensitivity for detecting recurrent tumour at the expense of specificity, while using cerebral cortex provides better specificity at the expense of sensitivity.
4. Lesion to reference region ratios using mean or maximum SUV can be used to provide a measure of FDG uptake in lesions. Numerical cut-off values for distinguishing tumour from benign lesions, such as radiation necrosis, and for grading tumours are not well established for FDG. If ratios are used, either white or grey matter rather than a mixture of the two should be used for reference due to the substantially higher FDG uptake in grey matter than in white matter. Additionally, normal-appearing brain should be considered for the reference region to provide consistency across studies.
5. The regional metabolic rate of glucose can be estimated in lesions and normal brain by compartmental modelling or by using graphical analytical approaches. A correction factor, the so-called “lumped constant” [30], can be used to convert the FDG values to values reflecting glucose metabolism [31]. However, little is known about the accuracy of these methods in brain tumours and/or treatment effects. Currently, there is insufficient data to recommend these types of quantitative studies in routine clinical FDG PET for neurooncology.

##### Cut-off thresholds for definition of biological tumour volume

- FDG: Not available
- FET: SUV >1.6–1.8 of the mean value in healthy appearing brain (*see* section Static FET, MET, FDOPA PET, item 4(a)) [32]
- MET: SUV >1.3 of the mean value in healthy appearing brain [33]
- FDOPA: SUV more than the mean value in healthy striatum [29] (not validated histologically)

##### Dynamic PET acquisition

An established clinical value for dynamic PET acquisitions applies only to FET [7]. Time–activity curves (TAC), i.e. the mean tissue radioactivity in the tumour region of interest (ROI)/VOI (SUV; becquerels per centimetre cubed, counts per centimetre cubed) as a function of time, can be generated from dynamic FET PET images. The TAC of the healthy brain (*see* section Static FET, MET, FDOPA PET, item 4(a)) should be plotted for comparison to exclude technical artefacts. To extract the TAC of the most aggressive tumour area and to provide sufficient count statistics for curve generation, the following approach is recommended:

1. The VOI is drawn semiautomatically using an individually adapted isocontour of the tumour maximum yielding a volume of 1–2 mL, or using a standard ROI/VOI with a fixed diameter of 1.6 cm centred on the tumour maximum yielding a volume of 2 mL.
2. These ROIs/VOIs can be defined on the summation images of 20–40 min or 10–30 min. The latter may be better suited to the depiction of the early peak uptake in more aggressive gliomas.
3. The shape of the TAC is classified as increasing, decreasing or plateau. For assessment of time to peak (TTP), the time of tumour peak uptake is noted.

##### Interpretation of static FET/MET/FDOPA PET data

Based on the 2016 WHO classification [34], gliomas will be progressively classified on the basis of histological and molecular characteristics, rather than as low-grade or high-grade gliomas, as follows:

1. Astrocytomas grade II and III (with isocitrate dehydrogenase 1 (IDH1) mutation, without 1p/19q codeletion) [35]
2. Oligodendrogliomas grade II and III (with IDH1 mutation, with 1p/19q codeletion) [36]
3. Wildtype astrocytomas or oligodendrogliomas not otherwise specified
4. Secondary glioblastomas (with IDH1 mutation)
5. Primary glioblastomas (IDH1 wildtype or IDH1-negative) [37, 38]

Available data on the range of radiotracer uptake are limited to FDG, FET and MET [39–43]. Further evidence is required to complement molecular characterization of gliomas and implement radiotracers in the new classification.

1. At primary diagnosis
  - Negative scan: uptake in the background uptake range or slightly above excludes a grade III/IV glioma, lymphoma or metastasis with high probability. Also, an oligodendroglial tumour is very unlikely. A grade I/II astrocytoma cannot be excluded since approximately 30% exhibit low uptake.
  - Positive scan: increased uptake has high a positive predictive value for a neoplastic process. A reliable differentiation of grade III/IV and grade I/II gliomas is not possible because of a high proportion of active tumours among the latter, especially in oligodendrogliomas. Local areas with the highest uptake should be used for biopsy guidance.
2. Therapy planning: for FET and MET, areas with uptake higher than the above biological tumour volume (BTV) cut-off activity values are used to delineate the metabolically active tumour tissue for planning of surgery and radiotherapy.
3. Tumour recurrence: increased uptake in the follow-up of previously treated glioma has high accuracy in differentiating treatment-related changes (e.g. pseudoprogression, radionecrosis) from recurrent disease.
4. Therapy monitoring: amino acid uptake progression during different kinds of therapy is indicative of therapy failure, while uptake regression indicates responsiveness. FET and FDOPA PET have been shown to identify pseudoresponse during antiangiogenic therapy.

The TBRmean and TBRmax thresholds for establishing pathological amino acid accumulation depends on the ROI definition technique, the spatial resolution of the PET scan (system type, reconstruction, data filtering) and the clinical question to be answered [44]. The thresholds according to clinical questions are shown in Table 1.

**Table 1:** Commonly used thresholds for amino acid PET, validated histologically or clinically, according to the clinical question

Clinical question	Tracer	Method		Threshold	Reference
Differentiation between neoplastic and non-neoplastic tissue	FET	TBRmax		2.5	[45]
		TBRmean		1.9	
	MET	TBRmax		1.3–1.5	[33, 46]
	FDOPA	–		n.a.	
Tumour grading (grade I/II versus III/IV glioma)	FET	TBRmean		1.9–2.0	[45, 47, 48]
		TBRmax		2.5–2.7	
		TTP		<35 min	
		TAC pattern (I, II, III)		Pattern II, III	
Tumour extent	FET	TBR		1.6	[32]
	MET	TBR		1.3	[49]
	FDOPA	TBR		2.0	[50]
Tumour recurrence	FET	TBRmean (circular ROI diameter 1.6 cm)		2.0	[51]
		TTP		<45 min	
	MET	TBRmax		1.6	[52]
	FDOPA	TSRmax		2.1	[53]
		TSRmean		1.8	
Malignant transformation of grade I/II glioma	FET	TBRmax		>33% increase	[54]
		TBRmean		>13% increase	
		TTP change in ROI >1.6 brain		6 min decrease	
Differentiation between *early* pseudoprogression and true progression	FET	TBRmax		2.3	[55]
Differentiation between *late* pseudoprogression and true progression	FET	TBRmax		1.9	[56]
		TBRmean		1.9	
Identification of responders in treatment response evaluation	FET	Radiochemotherapy (7–10 days)	TBRmax	>20% decrease	[57–59]
			TBRmean	>5% decrease	
		Bevacizumab/irinotecan (4–12 weeks)	BTV	>45% decrease	
	MET	Temozolomide	TBRmax	Stable or decreasing	[60]
	FDOPA	Bevacizumab (2 weeks)	BTV	>35% decrease	[29]
				<18 mL	

*TBR* tumour to background ratio, *TTP* time to peak, *TAC* time–activity curve, *TSR* tumour to striatum ratio, *ROI* region of interest

##### Interpretation of static FDG PET data

1. At primary diagnosis
  - Increasing FDG uptake by gliomas is correlated with higher grade and worse prognosis [7, 61–64]. Grade I/II gliomas typically have FDG uptake similar to or less than white matter uptake, although some grade I/II gliomas such as pilocytic astrocytomas have high FDG uptake. Grade III/IV gliomas typically have FDG uptake greater than white matter uptake (see comments on the new WHO classification scheme (*see* section Interpretation of static FET/MET/FDOPA PET data, item 1).
  - There is overlap in FDG uptake between grade I/II and grade III/IV gliomas, particularly for gliomas with FDG uptake greater than white matter but less than grey matter uptake. Optimal quantitative thresholds and visual analysis criteria have not been established for definitively distinguishing glioma grade or predicting prognosis based on FDG PET alone.
2. Tumour recurrence
  - High FDG uptake in enhancing brain lesions is correlated with tumour recurrence. However, high FDG uptake can occur in the brain after radiation therapy including radiation necrosis, and recurrent tumours may have relatively low FDG uptake. Well-defined quantitative and qualitative criteria with high diagnostic accuracy are not available and may not be achievable with FDG PET.
  - For gliomas treated with radiation therapy, FDG PET can be used to distinguish radiation necrosis from recurrent tumour. Many criteria have been proposed, and wide ranges of sensitivity and specificity have been reported in the literature [65–71]. A reasonable approach is to use normal white and grey matter as the reference regions. Lesions with FDG uptake similar to or less than white matter uptake are probably radiation necrosis while lesions with FDG uptake higher than grey matter uptake are probably recurrent tumour. Lesions with FDG uptake higher than white matter but less than grey matter uptake may be radiation necrosis, recurrent tumour or a mixture of both. Correlation with MRI, the presence of focal uptake suggesting recurrence within a larger region of diffuse lower level FDG uptake, and the clinical presentation may be useful in these cases.
  - The choice of the quantitative cut-off value or visual reference region will affect the sensitivity and specificity of the results. For example, using normal white matter as the reference region and categorizing lesion with FDG uptake similar to or less than white matter uptake as radiation necrosis and lesions with uptake higher than normal white matter uptake as recurrent tumour will provide higher sensitivity (negative imaging more likely to be treatment effect) at the expense of specificity (positive imaging more likely to be false positive). Similarly, using normal grey matter as the reference region will provide lower sensitivity (negative imaging more likely to be false-negative) with an increase in specificity (positive imaging more likely to be recurrent tumour).

##### Interpretation of dynamic FET PET data

1. An early peak in the TAC shape of the mean ROI/VOI activity (<20 min after injection) followed by a plateau or a decreasing TAC is indicative of a grade III/IV tumour.
2. Continuously increasing uptake up to 40 min after injection is more frequently observed in grade I/II gliomas, but is not specific. This TAC pattern is also typical of treatment-induced changes, e.g. radionecrosis, pseudoprogression.
3. A change in the TAC pattern during follow-up of grade I/II gliomas from an increasing TAC to an early peak with a decreasing TAC is indicative of malignant transformation [54].

##### Physiological tracer distribution

- FDG
  1. Common: high physiological uptake in grey matter (e.g. cerebral and cerebellar cortex, deep grey nuclei).
  2. Common: moderate uptake in the extraocular muscles.
  3. Occasionally: brain activation during the uptake phase such as patient motion or visual stimulation can result in higher uptake in the associated regions in the cerebral cortex.
- FET, MET [72]
  1. Common: slight uptake in vascular structures, basal ganglia, cerebellum, skin and salivary glands.
  2. Occasionally: slight focal uptake in pineal body, choroid plexus and clivus bone marrow.
- FDOPA
  1. Common: moderately increased uptake in basal ganglia and pituitary, and slight uptake in the cerebellum, skin, optic nerve, ocular muscles and salivary glands.
  2. Occasionally: pineal body.
  3. No increased uptake in vascular structures.

##### Known pitfalls

- All tracers
  1. Uptake may be increased in inflammatory lesions and epileptic seizures.
  2. Uptake may be underestimated in small lesions relative to image resolution.
- FDG
  1. High uptake in grey matter can obscure lesions within or adjacent to grey matter.
  2. High blood glucose levels at the time of injection decrease uptake in tumour and healthy appearing grey matter, but may not affect lesion detection.
  3. Perivascular infiltration.
  4. Anatomical abnormalities.
  5. Treatment effects may decrease uptake in the treatment area and brain regions that receive synaptic input from the treated area (diaschisis).
- MET, FET, FDOPA
  1. TBRmean, TBRmax and BTV may be overestimated if there is reduced uptake in the reference brain tissue VOI because of structural changes, e.g. atrophy, trauma and infarcts, or reduced tracer delivery. e.g. ischaemia.
  2. As MET, FET, and FDOPA are all transported across the blood–brain barrier and into cells by system L amino acid transport, they can all be expected to have similar pitfalls (Table 2).
- Dynamic FET
  1. In early images, up to 15 min after injection, blood pool uptake is relatively high, and tracer activity in vascular structures may have the same appearance as tumour tissue uptake.
  2. Reduced uptake in occipital and temporal skin areas may be seen in static images, probably as a result of reduced perfusion caused by the head padding.
  3. An increasing TAC may indicate inflammatory lesions.
  4. A decreasing TAC may be seen in WHO grade II oligodendroglial tumours (around 50%).
  5. A decreasing TAC may be seen in tumours close to sinuses because of the influence of venous blood activity.
  6. After antiangiogenic treatment the TAC may change from a decreasing to an increasing pattern.

**Table 2:** Known pitfalls and estimated occurrence of false-positive presentation in amino acid PET and FDG PET

Condition	Tracer	Estimated occurrence of false positive presentation	Reference
Brain abscess, infection, inflammation	MET	Rare	[73]
	FET	Rare	[58, 74, 75]
	FDG	Variable	[76]
Haematoma	MET	Increased uptake up to 45 days after bleeding	[46, 77, 78]
	FET	Increased uptake up to 14 days after bleeding	[79]
	FDG	Increased uptake up to 4 days after bleeding	[80]
Infarction	MET		[46, 81]
	FET	Increased uptake up to 7 days after ischaemia	[58, 82]
	FDG	Increased uptake up to 14 days after ischaemia	[80]
Developmental venous anomalies	FET	Rare	[83]
	FDOPA		[58, 82, 84]
Demyelination	MET	Rare	[33, 46]
	FET		[58, 74]
	FDG		[85]
Radionecrosis/radiation-induced changes	MET	Rare	[86]
	FET	Higher incidence of PET-positive findings in radiation-induced lesions during the first 6 months after focused high-dose radiotherapy	[87]
	FDG	Variable	[65]
Epileptic seizures	FET	Very rare	[88]
	MET		[89]
	FDG	Rare	[90]

## Documentation and reporting

Description of findings in brain tumour imaging should generally comply with previously published guidelines for FDG imaging in oncology and with regard to general aspects of reporting such as due diligence [6].

The content of the report affects patient management and clinical outcomes, and is a legal document. It is good practice to provide a structured report with concise concluding statements intended to answer the specific clinical question(s) posed, if possible.

Regardless of the radiotracer, reports should follow the general structure outlined below.

### General information

1. Name of the patient and other identifiers, such as date of birth.
2. Name of the referring physician.
3. Type and date of examination.
4. Radiopharmaceutical including route of administration and amount of activity administered.
5. Patient history with emphasis on diagnosis and tumour-related treatment and the clinical question leading to the study request (*see* sections Common clinical indications and Request).

### Body of the report

1. Procedure description
  - Information on the imaging procedure (e.g. static or dynamic scan), and time between PET tracer injection and image acquisition.
  - If FDG is used, the measured blood glucose level at the time of injection.
  - If sedation is performed, type and time of medication in relation to the tracer injection.
  - If a low-dose CT scan is performed for attenuation correction, a statement such as “not performed for diagnostic purposes, not replacing diagnostic CT” could be added.
  - The use of a nonconventional system type (e.g. PET/MRI) should be mentioned.
2. Data quality
  - Abnormal tracer biodistribution.
  - CT-related artefacts, e.g. from metallic implants.
  - Poor compliance with fasting.
  - Any observed events that may adversely influence interpretation, e.g. head movements, seizure activity.
  - If FDG is used, increased blood glucose level.
3. Comparative data
  - PET images should be compared with morphological data, particular MRI data, whenever possible.
  - PET images should be compared with previous PET scans to evaluate the course of disease.
  - The type and date of comparative data should be noted before description of the imaging findings.
4. Description of findings
  - The normality of radiotracer uptake should be stated, whether normal or abnormal.
  - In case of abnormal findings, the location (with a correct anatomical description), extent and intensity of pathological tracer accumulation in relation to normal tissue uptake should be described.
  - Uptake characteristics, including:
    Shape of uptake, e.g. focal, diffuse, inhomogeneous.
    Intensity of uptake in relation to healthy brain uptake (slight, moderate or strong).
    Extent and peak uptake, in correlation with, for example, T1 contrast enhancement and/or T2/FLAIR hyperintensity on MRI or obvious anatomical abnormalities on CT/low-dose CT images.
  - Semiquantitative parameters
    Calculate and report the TBRmax. Reporting the TBRmean and BTV is optional. Determination of FDG SUV/SUV ratio, TBRmean, TBRmax and BTV are optional in the clinical setting and do not have well-established utility in characterizing gliomas or distinguishing treatment effects from recurrence.
    When dynamic imaging is performed with FET, the pattern of the tumour TAC (increasing, decreasing, plateau) should be described. The reporting of TTP and slope is optional.
  - Clinically relevant incidental findings should be reported, e.g. extracerebral metastases.
  - Comparison with previously performed PET studies, e.g. for therapy response or malignant transformation.
5. Limitations:
  when appropriate, factors that could have limited data quality or diagnostic accuracy should be mentioned (Table 2).

### Interpretation

The interpretation should address the question raised in the clinical request and integrate medical history, comparative imaging and any limitations. A precise diagnosis should be given whenever possible. Additional scans or follow-up scans should be recommended when appropriate.

## Equipment specifications

### System specifications

The use of state-of-the-art 3D PET/CT or PET/MRI systems is recommended. The system should allow collection of low-dose CT images or MRI-based sequences that can be used for attenuation and scatter correction of the PET emission data. A dedicated brain PET-only system may be used provided it is equipped with transmission scan sources of sufficient strength – as recommended by the manufacturer – to ensure sufficient quality of the transmission scans and thereby of the PET emission data attenuation correction. PET(/CT) systems should have a minimal axial field of view of 15 cm to ensure sufficient coverage of the entire brain, including cerebellum and brainstem.

### PET acquisition

The system should be able to acquire both static and dynamic or list-mode PET emission data in 3D mode. Data should be reconstructed online or offline (i.e. retrospectively) in single or multiple frames, as specified by the study protocols and these guidelines. In addition, PET images should be able to be reconstructed with and without attenuation correction. The PET images without attenuation correction should not be used for primary interpretation but can be useful for recognizing attenuation artefacts in the attenuation-corrected PET images. The system should have all functionalities and methods available as required for quantitative brain PET imaging and reconstruction, including, but not limited to, online randoms correction, scatter correction, attenuation correction, dead time correction, decay and abundance correction and normalization (correction for detector sensitivities).

## Quality control and improvement

### Quality control and interinstitutional PET system performance harmonization

Various factors affecting PET image quality and quantification have previously been reviewed [91]. Although this review focused on the use of radiolabelled amino acids and FDG for glioma imaging, the technical and imaging physics uncertainties indicated in that review are valid for any PET examination regardless of radiotracer or specific application. The use of brain PET examinations in multicentre studies and/or when data are compared with a reference database or disease pattern, it is of the utmost importance that PET data are collected in such a manner that they can be pooled and compared. In order to guarantee sufficient image quality, quantitative performance and image harmonization, the performance of the PET systems must be regularly checked by several QC experiments. All regular and vendor-provided maintenance and QC procedures should be followed. QC experiments should at least address the following:

- Daily check of detector performance, i.e. with point, rod or cylindrical sources to automatically test and visualize the proper functioning of detector modules including inspection of 2D sinograms.
- Daily check of PET activity concentration measurement calibration using an activity filled cylindrical phantom source following the procedure recommended by the manufacturer.
- Cross-calibration of the PET(/CT) system against the locally used dose calibrator to prepare and measure patient-specific radiotracer activities. Cross-calibrations should be performed following EARL recommendations and criteria.
- Correct alignment between PET and CT should be verified according to the procedure and frequency recommended by the manufacturer.
- Additional QC procedures performed less frequently following the instructions provided by the manufacturer and the EANM recommendations for routine QC of nuclear medicine equipment [92].

### CT quality control

The EU guidelines for FDG PET/CT tumour imaging discuss several documents and reports that have been published on CT quality control (CT-QC). For example, an overview of CT-QC is given in the “Equipment Specifications” and “Quality Control” sections of the following American College of Radiology (ACR) practice parameters: “ACR–ASNR–SPR Practice parameter for the performance of Computed Tomography (CT) of the extracranial head and neck” (https://www.acr.org/-/media/ACR/Files/Practice-Parameters/CT-Head-Neck.pdf?la=en), “ACR–SCBT-MR–SPR–STR Practice parameter for the performance of thoracic computed tomography (CT)” (https://www.acr.org/-/media/ACR/Files/Practice-Parameters/CT-Thoracic.pdf?la=en), and “ACR–SPR Practice parameter for the performance of thoracic Computed Tomography (CT) of the abdomen and Computed Tomography (CT) of the Pelvis”, (https://www.acr.org/-/media/ACR/Files/Practice-Parameters/CT-Abd-Pel.pdf?la=en). CT-QC is also the subject of the Institute of Physics and Engineering in Medicine (IPEM) report 91 “Recommended Standards for the Routine Performance Testing of Diagnostic X-Ray Systems”. In addition, CT performance monitoring guidelines are given in the American College of Radiology: ”ACR–AAPM Technical standards for diagnostic medical physics performance monitoring of Computed Tomography (CT) Equipment”, (https://www.acr.org/-/media/ACR/Files/Practice-Parameters/CT-Equip.pdf?la=en).

### MR quality control in PET/MRI

While there are no regulatory requirements for special/standard QC/QA procedures for MRI systems, numerous points should be considered for conducting safe and high-quality MRI examinations as outlined in section Examination procedures/specifications. It is advisable to adhere to the recommendations of the manufacturer (i.e. follow the planned maintenance intervals). For PET/MRI systems an approach to basic MRI QC to be performed by the user is described in a review by Sattler et al. [93].

## Radiation safety

The systemic use of radiotracers leads to systemic exposure of the patients to radiation. The amounts of radioactivity usually delivered with the administration of ^11^C-labelled and ^18^F-labelled PET radioligands (*see* section Administered activity in adults) result in effective doses (ED) of the same order of magnitude as delivered by other ^11^C-labelled and ^18^F-labelled radiotracers [94]. The radiation dose from low-dose CT scans of the head region depends on the CT scanning parameters and is generally well below 0.5 mSv. The overall ED from PET/CT investigations of the head region, when accounting for the whole-body exposure, should remain near or below 5 mSv. In adults, the organ with the highest radiation dose for all the above tracers is the urinary bladder wall (Table 3).

**Table 3:** Radiation dosimetry

Radiotracer	Adult organ with highest dose (mGy/MBq)	Effective dose (mSv/MBq)		Reference
		Adult	Paediatric	
^18^F-FDG	Urinary bladder wall: 0.13	0.019	1 year: 0.095 5 years: 0.056 10 years: 0.037 15 years: 0.024	[95]
^18^F-FET	Urinary bladder wall: 0.085	0.016	1 year: 0.082 5 years: 0.047 10 years: 0.031 15 years: 0.021	[95]
^11^C-MET	Urinary bladder wall: 0.092	0.0082	1 year: 0.047 5 years: 0.025 10 years: 0.016 15 years: 0.011	[95]
^18^F-FDOPA	Urinary bladder wall: 0.30	0.025	1 year: 0.10 5 years: 0.07 10 years: 0.049 15 years: 0.032	[95]

## Conclusion

Since the previous EANM guidelines in 2006 [10], the clinical use of molecular imaging with PET and PET/CT in the diagnosis of glioma has continuously increased in Europe and the US. For successful and appropriate use of this technology, a clear understanding of the capabilities and limitations of the technology and appropriate patient selection, preparation, scan acquisition and image reconstruction are required. This document attempts to provide some guidance on the performance and interpretation of molecular imaging to supplement recent clinical guidelines [7], and to bring PET brain imaging into daily clinical practice and into larger scale interinstitutional clinical neurooncological trials across imaging platforms.
